# Design, Synthesis, and Biological Evaluation of Novel PARP-1 Inhibitors Based on a 1*H*-Thieno[3,4-*d*] Imidazole-4-Carboxamide Scaffold

**DOI:** 10.3390/molecules21060772

**Published:** 2016-06-13

**Authors:** Lingxiao Wang, Feng Liu, Ning Jiang, Wenxia Zhou, Xinbo Zhou, Zhibing Zheng

**Affiliations:** 1 Laboratory of Computer-Aided Drug Design & Discovery, Beijing Institute of Pharmacology and Toxicology, Beijing 100850, China; momo9060@163.com; 2 Department of Neuroimmunopharmacology, Beijing Institute of Pharmacology and Toxicology, Beijing 100850, China; pharmacare@126.com (F.L.); jiangning@bmi.ac.cn (N.J.); zhouwx@nic.ac.cn (W.Z.)

**Keywords:** PARP-1 inhibitor, BRCA1/2, anti-tumour, thieno[3,4-*d*]imidazole

## Abstract

A series of poly(ADP-ribose)polymerase (PARP)-1 inhibitors containing a novel scaffold, the 1*H*-thieno[3,4-*d*]imidazole-4-carboxamide moiety, was designed and synthesized. These efforts provided some compounds with relatively good PARP-1 inhibitory activity, and among them, **16l** was the most potent one. Cellular evaluations indicated that the anti-proliferative activities of **16g**, **16i**, **16j** and **16l** against BRCA-deficient cell lines were similar to that of olaparib, while the cytotoxicities of **16j** and **16l** toward human normal cells were lower. In addition, ADMET prediction results indicated that these compounds might possess more favorable toxicity and pharmacokinetic properties. This study provides a basis for our further investigation.

## 1. Introduction

Poly(ADP-ribose)polymerases (PARPs) are nuclear enzymes that play important roles in the genomic repair process [1,2]. PARP-1, the most abundant and best-characterized member of the PARP superfamily [3], has emerged as a promising molecular target for the treatment of cancer in the past decade [4]. When activated by DNA damage, it catalyzes the transfer of ADP-ribose units to target proteins (using nicotinamide adenine dinucleotide (NAD^+^) as a substrate) to facilitate DNA repair. It is a key step in the base excision repair (BER) of single-strand DNA breaks (SSB) [5], which are involved in the resistance that often develops after traditional cancer therapies. Therefore, combination of PARP-1 inhibitors with radio- and chemo-therapy to enhance antitumor effects has been an initial focus in cancer treatment [6]. However, this strategy has been hampered by enhanced toxicity and has not succeeded despite 30 years of research [7].

**Figure 1:**
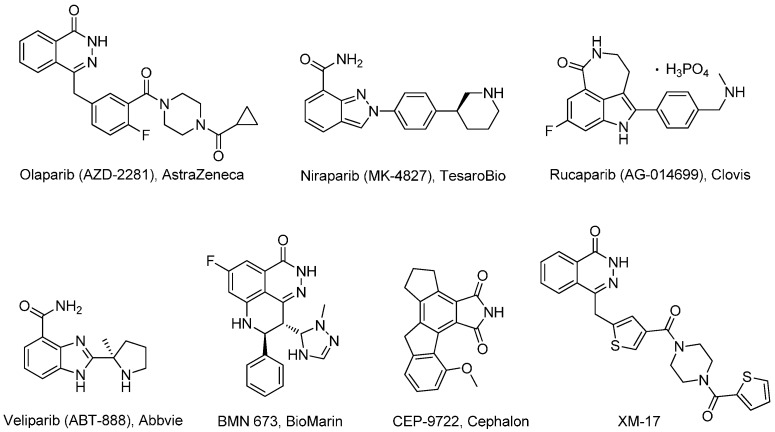
Structures of PARP-1 inhibitors.

Recently, PARP-1 inhibition has been demonstrated as an effective method for inducing synthetic lethality in cancers that have defective homologous recombination repair (HRR) pathway, such as cancers with BRCA-1/2 mutation [8,9]. For such cancers, PARP-1 inhibitors are emerging as promising monotherapy agents [10]. This strategy has facilitated the approval of olaparib (Figure 1) for advanced and relapsed ovarian cancer with germline BRCA mutations. A number of PARP-1 inhibitors have been discovered and several of them are in different stages of clinical trials, including veliparib [11], rucaparib [12] and niraparib (Figure 1). This brings hope for the treatment of other advanced refractory cancers, such as triple-negative breast, pancreatic, and prostate cancers [13,14,15]. Furthermore, the latest studies have shown that the antitumor effects of PARP-1 inhibitors were enhanced by combination with other agents, such as AKT inhibitors, C-Met inhibitors and PI3K inhibitors [16,17,18]. These findings have broadened the application area and increased the therapeutic potential of PARP-1 inhibitors. However, as the first on-market PARP-1 inhibitor [19], Olaparib is being used clinically at a high dose (400 mg twice-daily, 16 capsules) [20], and still has some deficiencies such as potential toxicity [21] and inadequate pharmacokinetic properties. Thus, efforts pursuing new inhibitors are still needed.

**Figure 2:**
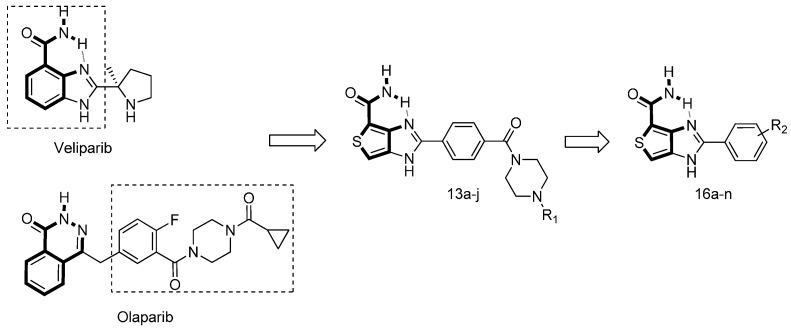
The design of novel PARP-1 inhibitors containing a thienoimidazole scaffold.

The design of PARP-1 inhibitors is usually based on the nicotinamide moiety of NAD^+^ to mimic the substrate-protein interaction of NAD^+^ with enzyme [22]. According to previous literatures [23,24], despite the large variety in chemical structures of current PARP-1 inhibitors, the common structural features are aromatic ring and carboxamide moiety, which are responsible for the formation of hydrogen bonds and pi-stacking interaction with PARP-1, respectively. On this basis, we have previously reported a series of potent phthalazinone-containing PARP-1 inhibitors with fairly good potency [25], such as XM-17 (Figure 1). In this study, we report a series of compounds containing a novel thieno-imidazole scaffold that can form a six-membered ’pseudo-cycle’ through an intramolecular hydrogen bond (Figure 2). Our preliminary efforts identified **16l** as the best potential compound that displayed comparable enzymatic and anti-proliferative potency, lower cytotoxicity, and better predicted ADMET properties compared to olaparib.

## 2. Results and Discussion

### 2.1. Chemistry

**Scheme 1:**
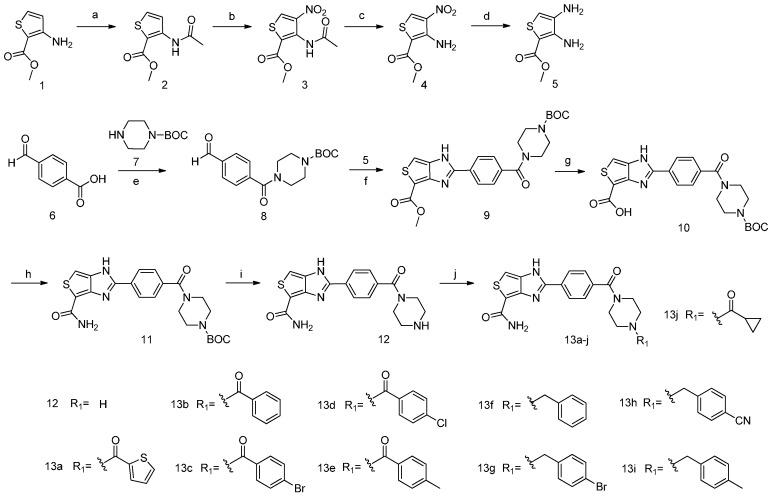
Synthetic route for target compounds **13a**–**j**. *Reactions and conditions*: (**a**) (CH_3_O)_2_CO; (**b**) HNO_3_, H_2_SO_4_, −25 °C; (**c**) CH_3_ONa, CH_3_OH; (**d**) Pd/C, CH_3_OH; (**e**) EDCI, HOBt, DIPEA, DCM; (**f**) i) 1,4-dioxane; ii) iodobenzene diacetate, 55 °C; (**g**) 4N NaOH, CH_3_OH; (**h**) i) CDI, DMF; ii) NH_3_·H_2_O; (**i**) TFA; (**j**) TEA or K_2_CO_3_, DMF.

Synthesis of compounds **13a**–**j** is outlined in Scheme 1. Briefly, acetylation of carboxylate **1** was followed by nitration with a mixture of concentrated nitric and sulphuric acids to obtain compound **3**. Deprotection of the acetyl group with NaOCH_3_ furnished amine **4** that was converted to the key intermediate **5** by catalytic hydrogenation in the presence of Pd/C. Subsequently, acid **6** was coupled with piperazine **7** under standard amidation conditions (EDCI, HOBT, DIPEA), which yielded amide **8**. Cyclization of the intermediate **5** with **8** in the presence of iodobenzene diacetate provided ester **9**. Saponification of the ester **9** followed by ammonolysis provided compound **11**. Deprotection of the BOC-group with trifluoroacetic acid yielded a secondary amine **12**. Finally, amine **12** was coupled with various benzyl bromides or acyl chlorides to obtain the corresponding target compounds **13a**–**j**.

**Scheme 2:**
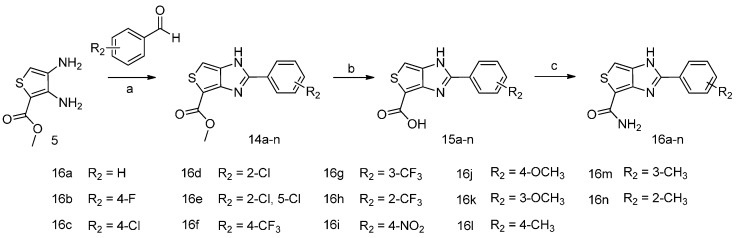
Synthetic route for target compounds **16a**–**n**. *Reactions and conditions*: (**a**) i) 1,4-dioxane; ii) iodobenzene diacetate, 55 °C; (**b**) 4N NaOH, CH_3_OH; (**c**) i) CDI, DMF; ii) NH_3_·H_2_O.

Synthesis of compounds **16a**–**n** is shown in Scheme 2. Cyclization of the intermediate **5** with substituted benzaldehydes in the presence of iodobenzene diacetate provided intermediates **14a**–**n**. Hydrolysis of esters **14a**–**o** provided intermediates **15a**–**n**. Lastly, ammonolysis furnished imidazo [3,4-*d*]thienocarboxamide derivatives **16a**–**n**.

### 2.2. Biological Evaluation

#### 2.2.1. PARP-1 Enzyme Inhibitory Activity

All target compounds were evaluated *in vitro* for their PARP-1 enzyme inhibitory activity. Compounds that showed >50% inhibition at 10 μM were selected to determine their IC_50_ values. The IC_50_ values of target compounds **12** and **13a**–**j** against PARP-1 enzyme are shown in Table 1. 

Unfortunately, all target compounds showed limited activity (IC_50_ 0.723–3.864 μM) compared to the control compounds. The *ortho*-thenoyl- and benzoyl-substituted compounds **13a** and **13b** showed weaker inhibitory effects than veliparib and olaparib. Introduction of electron-withdrawing and donating groups on the benzene ring (compounds **13c**–**e**) did not elicit significant changes in the inhibitory activity. Similarly, there was no change in the activity of substituted benzyl compounds **13f**–**i**. However, the cyclopropyl substituted compound **13j** showed better activity than the aryl-substituted compounds. Interestingly, compound **12** without substitution proved to be the most potent one. This indicates that the large piperazine side chains could not be tolerated on the thienoimidazole scaffold. In addition, it indicates that higher potency of the compounds was associated with smaller size of the R_1_ substituents. Based on this structure-activity relationship, a series of compounds **16a**–**n** without the piperizine groups were designed and synthesized. 

IC_50_ values of compounds **16a**–**n** against PARP-1 enzyme are shown in Table 2. Most of these compounds showed better activity than compounds **13a**–**j** due to the removal of the large piperazine side chains. The most potent compound was **16l** that showed an IC_50_ value of 0.043 μM, which is only four times less potent than the reference compound. Introduction of an electron-withdrawing or donating group at the phenyl *para* position contributed to the activity of compounds (**16b**, **16c**, **16f**, **16i**, **16j**, **16l** *vs.* **16a**). Compounds featuring substitutions at the *para* position were generally more active than the corresponding analogues containing substitutions at the *meta* or *ortho* position (**16c** *vs.* **16d**, **16f** *vs.* **16h**, **16j** *vs.* **16k**, **16l** *vs.* **16m** and **16n**), with only one exception (**16f** *vs.* **16g**).

#### 2.2.2. Anti-Proliferative Activity on BRCA1/2-Deficient Cell Lines

In 2005, two landmark studies [26,27] were published and revealed that PARP-1 inhibitors were synthetically lethal in tumor cells with BRCA-1/2 mutation, which led to a major interest in this class of drug. Synthetic lethality means that when PARP-1 inhibitors suppress the role of BER pathway, the unrepaired SSBs would accumulate and collapse the DNA replication forks to form potentially lethal DSBs, and the normal cells would survive by repairing these DSBs by HRR pathway in this case, but in BRCA-deficient tumor cells, unrepaired DSBs would result in cell death. Studies showed that over 20% of high-grade serous ovarian cancers exhibited germline or somatic mutations in this gene [28].

HCC1937 (with BRCA1 mutation) and CAPAN-1 (with BRCA2 mutation) human tumor cell lines are commonly used for evaluating the anti-proliferative potencies of PARP-1 inhibitors as monotherapy *in vitro*. Preliminary PARP-1 enzyme assay identified some compounds with good inhibitory activities. The four most active compounds (**16g**, **16i**, **16j**, and **16l**) were selected and their *in vitro* anti-proliferative activity against HCC1937 and CAPAN-1 cell lines was further evaluated (Table 3). Interestingly, the four compounds displayed similar or improved activities compared with control compounds. Especially on the BRCA-1 deficient HCC1937 cell line, four compounds displayed 2.5~3.8-fold better activities than olaparib. 

The difference in cellular activities might be due to the different membrane permeability, and we tried to explain it through ADMET properties prediction preliminarily (Table 4). Further investigations into their biological activities and mechanism of action are underway.

#### 2.2.3. Cytotoxicity Assay on Normal Human Lung Fibroblast (HLF) Cells

Given the good results on BRCA1/2-deficient cell lines, the four compounds **16g**, **16i**, **16j**, **16l** were further preliminarily assayed for their cytotoxicity on normal human cells. As shown in Table 5, among these compounds, **16j** and **16l** showed lower cytotoxicity with IC_50_ values greater than 300 µM, and the inhibition rate (32.37%, 27.94%) was far lower than olaparib and veliparib (91.45%, 93.64%) at the concentration of 300 µM.

### 2.3. ADMET Prediction

We further predicted the ADMET properties of compound **16g**, **16i**, **16j** and **16l** using ADMET Predictor 7.0 (Simulations Plus, Inc., WestLancaster, CA, USA, 2014) to preliminarily validate their druggability. This computer program has been consistently ranked as the most accurate, quick and useful tool to predict the crucial physicochemical and biological properties for candidate compounds [29]. The selected ADMET properties of compounds are listed in Table 4, with emphasis on the absorption, metabolism and toxicity properties. ADMET_risk means the overall evaluation of compounds by ADMET predictor 7.0, and it demonstrated that **16j** and **16l** had better ADMET properties than olaparib (1, 1 *vs.* 4.85). In detail, **16j** and **16l** are predicted to have better oral absorption properties than olaparib, see in Sw, FaSSGF, logP and Peff values; Toxicity prediction indicated that the toxic risk of **16j** and **16l** are smaller than that of Olaparib, and this result is correspond with the cytotoxicity assay on HLF cells. It appears that the compounds with electron-donating groups (**16j**, **16l**) possess better ADMET properties than those with electron-withdrawing groups (**16g**, **16i**).

### 2.4. Molecular Docking

**Figure 3:**
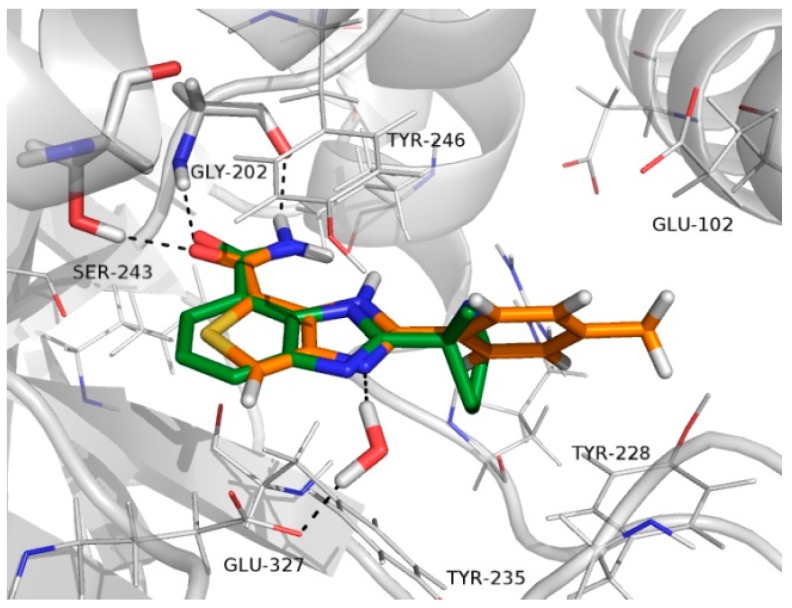
Proposed binding mode of compound **16l** overlaid with the X-ray co-crystal structure of Veliparib. Key amino acids are depicted as sticks and the atoms are coloured as carbon-grey, hydrogen-grey, nitrogen-purple and oxygen-red. Ligands are distinguished by differently coloured carbon atoms; Veliparib coloured as carbon-green and compound **16l** coloured as carbon-orange.

**Figure 4:**
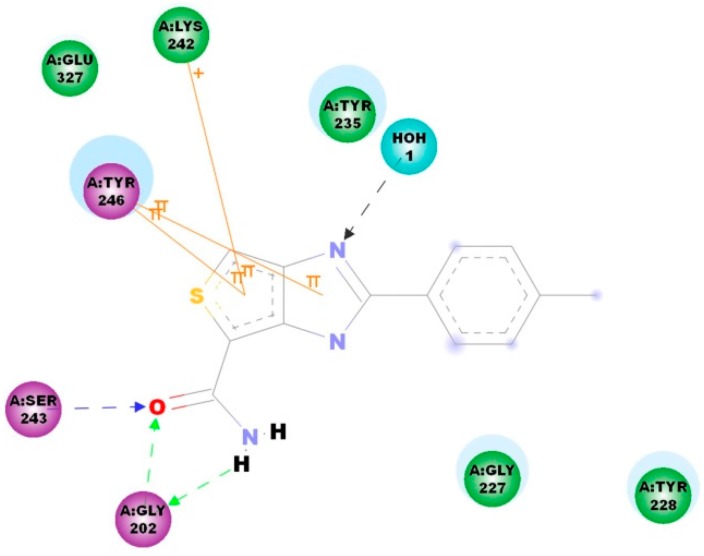
2D diagram of compound **16l** docking in the catalytic domain of human PARP-1 (PDB code: 2RD6).

In order to validate the results obtained from enzyme inhibition assay, a docking study was performed for compound **16l** and the catalytic domain of human PARP-1 (PDB code: 2RD6), as shown in Figure 3 and Figure 4. 

Consistent with previous reports, three key hydrogen-bonding interactions between the carboxamide group of **16l** with Gly-202 and Ser-243 were observed. Moreover, 1-NH of the thienoimidazole ring appeared to have formed a water-mediated hydrogen bond with Glu-327. Thienoimidazole ring exhibited a characteristic p-stacking interaction with Tyr-246. Figure 3 shows an X-ray co-crystal structure of veliparib overlaid with compound **16l** in PARP-1 catalytic domain. Both compounds demonstrated similar conformation in the active site. The carboxamide group achieved an optimal orientation through an intramolecular hydrogen bond for interacting with the key amino acids.

## 3. Experimental Section

### 3.1. General

All reagents and solvents were used as received from commercial sources. All reactions were monitored by thin-layer (TLC). Melting points (m.p., °C, uncorrected) were determined in open glass capillaries with an YRT-3 (Tianda Tianfa Technology Co., Ltd., Tianjin, China) electrothermal melting point apparatus. The ^1^H-NMR and ^13^C-NMR spectra were recorded in DMSO-*d*_6_ on a JNM-ECA-400 400 MHz spectrometer (JEOL Ltd., Tokyo, Japan), and chemical shifts are expressed as δ values relative to TMS as internal standard. ESI spectra (positive ion mode) were recorded on an API 3000 triple-quadrupole mass spectrometer (AB Sciex, Concord, ON, Canada).

### 3.2. Chemical Synthesis

#### 3.2.1. Synthesis of Compounds **13a**–**j**

*Methyl 3-acetamidothiophene-2-carboxylate* (**2**). Methyl 3-aminothiophene-2-carboxylate (**1**, 50.0 g, 318.09 mmol) was added portionwise to acetic anhydride (600 mL) and stirred at room temperature for 6 h. The mixture was then poured into cold water and white precipitate was generated. Sodium hydroxide was added until the acetic anhydride layer disappeared. The white solid was filtrated and washed with water (50.72 g, 80.0%). ESI-MS *m*/*z*: 200.2 [M + H]^+^, 223.3 [M + Na]^+^. ^1^H-NMR (DMSO), δ (ppm): 9.99 (s, 1H), 7.92 (d, *J* = 7.8 Hz, 1H), 7.87 (d, *J* = 7.8 Hz, 1H), 3.83 (s, 3H), 2.16 (s, 3H).

*Methyl 3-acetamido-4-nitrothiophene-2-carboxylate* (**3**). A solution of **2** (15.0 g, 75.29 mmol) in 95%–98% sulfuric acid (150 mL) was cooled to −30 °C. Then 65%–68% nitric acid (10 mL) was added dropwise, and the temperature was controlled under −20 °C. After reaction, the mixture was poured into 800 mL ice water. The yellow solid was filtrated and washed with water. The crude was purified through recrystallization in dichloromethane to afford the title compound as a white solid (5.5 g, 29.8%). ESI-MS *m*/*z*: 245.2 [M + H]^+^, 267.2 [M + Na]^+^. ^1^H-NMR (DMSO), δ (ppm): 10.26 (s, 1H), 8.61 (s, 1H), 3.85 (s, 3H), 2.06 (s, 3H).

*Methyl 3-amino-4-nitrothiophene-2-carboxylate* (**4**). Sodium methoxide (8.1 g, 149.9 mmol) was added portionwise to a solution of **3** (24.4 g, 99.9 mmol) in CH_3_OH (600 mL) and stirred at 55 °C for 8 h. After reaction, the mixture was poured into cold water and the yellow product was collected by filtration without further purification (19.5 g, 96.5%). ESI-MS *m*/*z*: 203.1 [M + H]^+^, 225 [M + Na]^+^. ^1^H-NMR (DMSO), δ (ppm): 8.55 (s, 1H), 7.18 (s, 2H), 3.82 (s, 3H)

*Methyl 3,4-diaminothiophene-2-carboxylate* (**5**). 10% Pd/C (2.0 g) was added to a solution of **4** (20.2 g, 100 mmol) in CH_3_OH (300 mL). Hydrogen was passed over to 4 bar pressure and the mixture was stirred at 40 °C for 8 h. After reaction, the Pd/C was filtered off and the organic phase was concentrated *in vacuo* to give the title compound as a yellow solid (15.6 g, 90.7%). ESI-MS *m*/*z*: 173.2 [M + H]^+^. ^1^H-NMR (DMSO), δ (ppm): 7.05 (s, 1H), 6.56 (s, 2H), 6.35 (s, 2H), 3.88 (s, 3H).

*tert-Butyl 4-(4-formylbenzoyl)piperazine-1-carboxylate* (**8**). 4-Formylbenzoic acid (**6**, 3.0 g, 10.0 mmol) was added into a 250 mL round-bottom flask and dissolved in 100 mL DCM. EDCI (2.3 g, 12.0 mmol), HOBt (0.013 g, 0.1 mmol), DIPEA (1.3 g, 10.0 mmol), N-BOC piperazine **7** (1.9 g, 10.1 mmol) was added successively and the mixture was stirred at room temperature overnight. After reaction, the mixture was washed with 1N NaOH, 1N HCl, brine, and then dried over Na_2_SO_4_. The organic was concentrated *in vacuo* to give the title compound as a white solid (5.8 g, 91.2%). ESI-MS *m*/*z*: 319.4 [M + H]^+^. ^1^H-NMR (DMSO-*d*_6_), δ: 10.06 (s, 1H), 7.81 (dd, 4H), 3.62 (s, 2H), 3.43 (s, 2H), 3.32 (s, 2H), 3.26 (s, 2H), 1.41 (s, 9H).

*Methyl 2-(4-(4-(tert-butoxycarbonyl)piperazine-1-carbonyl)phenyl)-1H-thieno[3,4-d] imidazole-4-carboxylate* (**9**). Intermediate **5** (1.7 g, 10.1 mmol) and **8** (3.18 g, 10.0 mmol) was added into a 100 mL round- bottom flask and dissolved in 1,4-dioxane (35 mL). The mixture was stirred at room temperature for 2 h and then heated up to 55 °C. Iodobenzene diacetate (6.4 g, 20.0 mmol) was added and the mixture was reacted for another 5 min. Then the reaction was quenched with saturated Na_2_S_2_O_4_ and NaHCO_3_ solution. The mixture was extracted with 50 × 3 EA and the combined organic layer was washed with brine, dried over anhydrous Na_2_SO_4_ and purified by column chromatography (PE–EA = 5:1) to afford **9** as a white solid (0.85 g, 18.1%). ESI-MS *m/z*: 471.5 [M + H]^+^. ^1^H-NMR (DMSO-*d*_6_), δ: 12.79 (s, 1H), 8.34 (d, *J* = 8.3 Hz, 2H), 7.83 (s, 1H), 7.59 (d, *J* = 8.3 Hz, 2H), 3.88 (s, 3H), 3.52 (m, 8H), 1.42 (s, 9H).

*2-(4-(4-(tert-Butoxycarbonyl)piperazine-1-carbonyl)phenyl)-1H-thieno[3,4-d]imidazole-4-carboxylic acid* (**10**). Intermediate **9** (4.7 g, 9.9 mmol) was added into a 150 mL round-bottom flask and dissolved in CH_3_OH (75 mL), then 4N NaOH solution (10 mL) was added and the mixture was stirred at reflux overnight. After reaction, the pH was adjusted to 6–7 with 2N HCl. The resulting yellow solid was separated by filtration and used for the next step without further purification (4.2 g, 92.1%).

*tert-Butyl 4-(4-(4-carbamoyl-1H-thieno[3,4-d]imidazol-2-yl)benzoyl)piperazine-1-carboxylate* (**11**). Acid **10** (4.2 g, 9.2 mmol), CDI (1.5 g, 9.2 mmol) was dissolved in DMF (25 mL) and the mixture was stirred at room temperature for 2 h. Then the solution was added dropwise to aqueous ammonia (75 mL) and stirred overnight. The mixture was extracted with 100 mL × 3 EA and the combined organic layer was washed with brine, dried with anhydrous Na_2_SO_4_ and concentrated *in vacuo*. The crude product was recrystallized with CH_3_OH to give compound **11** as a white solid (3.2 g, 76.2%). ESI-MS *m*/*z*: 456.5 [M + H]^+^. ^1^H-NMR (DMSO-*d*_6_), δ: 12.51 (d, 1H), 8.35 (d, *J* = 8.3 Hz, 2H), 7.82 (s, 1H), 7.55–7.82 (m, 4H), 3.54 (m, 8H), 1.42 (s, 9H).

*2-(4-(Piperazine-1-carbonyl)phenyl)-1H-thieno[3,4-d]imidazole-4-carboxamide* (**12**). Compound **11** (3.2 g, 7.0 mmol) was dissolved in THF (45 mL) and TFA (10 mL) was added and stirred at room temperature. After reaction, the solvent was removed and diluted in 2N HCl. The solution was washed with EA and then adjusted the pH to 8–9 with 2N NaOH. The resulting precipitate was filtrated and recrystallized with CH_3_OH to give 12 as a yellow solid (2.2 g, 88.1%). m.p.: 286–289 °C. ^1^H-NMR (DMSO-*d*_6_), δ: 12.93 (s, 1H), 8.28 (d, *J* = 8 Hz, 2H), 7.77 (s, 1H), 7.48–7.97 (m, 3H), 3.56 (s, 4H), 3.38 (s, 4H), 1.93 (s, 1H). ^13^C-NMR (DMSO-*d*_6_), δ: 168.94, 162.69, 161.61, 159.37, 159.05, 137.39, 131.07, 128.08, 127.49, 118.77, 115.81, 42.79. ESI-HRMS: *m*/*z* [M + H]^+^, calculated for C_17_H_17_N_5_O_2_S: 356.1181; found: 356.1173.

*2-(4-(4-(Thiophene-2-carbonyl)piperazine-1-carbonyl)phenyl)-1H-thieno[3,4-d]imidazole-4-carboxamide* (**13a**). Compound **12** (0.20 g, 0.56 mmol), thiophene-2-carbonyl chloride (0.08 g, 0.56 mmol) was dissolved in DMF (5 mL). TEA (2 drops) was added and the mixture was stirred at room temperature overnight. Then the mixture was diluted with EA (100 mL) and washed with 30 mL × 3 water, brine and dried over Na_2_SO_4_. The solvent was removed and recrystallized with CH_3_OH to give **12a** as a white solid (0.085 g, 33.2%). m.p.: 246–260 °C. ^1^H-NMR (DMSO-*d*_6_), δ: 12.64 (s, 1H), 8.32–8.15 (m, 2H), 7.65 (dd, *J* = 16.9, 7.3 Hz, 4H), 7.51 (s, 1H), 7.38 (t, 3H), 3.67 (s, 4H), 3.44 (s, 4H). ^13^C-NMR (DMSO-*d*_6_), δ: 169.36, 168.60, 162.58, 161.81, 150.45, 139.00, 138.01, 135.66, 130.48, 129.78, 128.54, 127.82, 127.11, 47.24, 41.59. ESI-HRMS: *m*/*z* [M + H]^+^, calculated for C_22_H_19_N_5_O_3_S_2_: 466.1007; found: 466.1001.

*2-(4-(4-Benzoylpiperazine-1-carbonyl)phenyl)-1H-thieno[3,4-d]imidazole-4-carboxamide* (**13b**). The title compound was obtained similarly to **13a** as a white solid (0.096 mg, 37.2%). m.p.: 270–272 °C. ^1^H-NMR (DMSO-*d*_6_), δ: 12.60 (d, 1H), 8.25 (dd, *J* = 15.1, 6.1 Hz, 2H), 7.71 (s, 1H), 7.63 (d, *J* = 8.0 Hz, 2H), 7.33–7.51 (m, 7H), 3.69 (s, 4H), 3.43 (s, 4H). ESI-HRMS: *m*/*z* [M + H]^+^, calculated for C_24_H_21_N_5_O_3_S: 460.1443; found: 460.1437.

*2-(4-(4-(4-Bromobenzoyl)piperazine-1-carbonyl)phenyl)-1H-thieno[3,4-d]imidazole-4-carboxamide* (**13c**). The title compound was obtained similarly to **13a** as a yellow solid (110 mg, 36.3%). m.p.: 294–296 °C. ^1^H-NMR (DMSO-*d*_6_), δ: 12.79 (s, 1H), 8.26 (d, *J* = 7.9 Hz, 2H), 7.79 (d, *J* = 5.0 Hz, 2H), 7.64 (d, *J* = 8.1 Hz, 2H), 7.46 (d, *J* = 3.3 Hz, 2H), 7.36 (s, 2H), 7.18–7.09 (m, 1H), 3.74 (s, 4H), 3.48 (s, 4H). ESI-HRMS: *m*/*z* [M + H]^+^, calculated for C_24_H_20_BrN_5_O_3_S: 538.0548; found: 538.0543.

*2-(4-(4-(4-Chlorobenzoyl)piperazine-1-carbonyl)phenyl)-1H-thieno[3,4-d]imidazole-4-carboxamide* (**13d**). The title compound was obtained similarly to **13a** as a white solid (0.13 g, 46.8%). m.p.: 247–249 °C. ^1^H-NMR (DMSO-*d*_6_), δ: 12.62 (d, 1H), 8.24 (d, *J* = 6.9 Hz, 2H), 7.81 (d, *J* = 8.3 Hz, 2H), 7.66 (s, 1H), 7.56 (dd, *J* = 16.6, 8.3 Hz, 4H), 7.35 (d, *J* = 17.2 Hz, 2H), 3.64 (d, 4H), 3.38 (m, 4H). ESI-HRMS: *m*/*z* [M + H]^+^, calculated for C_24_H_20_ClN_5_O_3_S: 494.1053; found: 494.1051.

*2-(4-(4-(4-Methylbenzoyl)piperazine-1-carbonyl)phenyl)-1H-thieno[3,4-d]imidazole-4-carboxamide* (**13e**). The title compound was obtained similarly to **13a** as a white solid (0.13 g, 46.8%). m.p.: 308–310 °C. ^1^H-NMR (DMSO-*d*_6_), δ: 12.63 (d, 1H), 8.24 d, 2H), 7.69 (s, 1H), 7.63 (d, J = 8.3 Hz, 2H), 7.38 (m, 2H), 7.30 (dd, *J* = 28.9, 8.4 Hz, 4H), 3.56 (d, 8H), 2.34 (s, 3H). ESI-HRMS: *m*/*z* [M + H]^+^, calculated for C_25_H_23_N_5_O_3_S: 474.1600; found: 474.1601.

*2-(4-(4-Benzylpiperazine-1-carbonyl)phenyl)-1H-thieno[3,4-d]imidazole-4-carboxamide* (**13f**). Compound **12** (0.20 g, 0.56 mmol), bromomethyl benzene (0.10 g, 0.56 mmol) was dissolved in DMF (5 mL). K_2_CO_3_ (0.15 g, 1.1 mmol) was added and the mixture was stirred at room temperature overnight. Then the mixture was diluted with EA (100 mL) and washed with 30 mL × 3 water, brine and dried over Na_2_SO_4_. The solvent was removed and recrystallized with CH_3_OH to give **12a** as a white solid (0.12 g, 47.9%). m.p.: 285–288 °C. ^1^H-NMR (DMSO-*d*_6_), δ: 12.58 (d, 1H), 8.23 (d, *J* = 7.8 Hz, 2H), 7.68 (s, 1H), 7.64 (d, *J* = 8.3 Hz, 2H), 7.38 (m, 3H), 7.30 (m, 4H), 3.76 (s, 2H), 3.56 (d, 8H). ESI-HRMS: *m*/*z* [M + H]^+^, calculated for C_24_H_23_N_5_O_2_S: 446.1650; found: 446.1651.

*2-(4-(4-(4-Bromobenzyl)piperazine-1-carbonyl)phenyl)-1H-thieno[3,4-d]imidazole-4-carboxamide* (**13g**). The title compound was obtained similarly to compound **13f** as a yellow solid (0.14 g, 47.5%). m.p.: 285–288 °C. ^1^H-NMR (DMSO-*d*_6_), δ: 265 °C–266 °C ^1^H-NMR (DMSO-*d*_6_), δ: 12.82 (s, 1H), 8.26 (d, 2H), 7.32–7.66 (m, 9H), 3.33–3.64 (m, 8H), 3.95 (s, 2H). ESI-HRMS: *m*/*z* [M+H]^+^, calculated for C_24_H_22_Br N_5_O_2_S: 524.0756; found: 524.0758.

*2-(4-(4-(4-Cyanobenzyl)piperazine-1-carbonyl)phenyl)-1H-thieno[3,4-d]imidazole-4-carboxamide* (**13h**). The title compound was obtained similarly to compound **13f** as a white solid (0.09 g, 34.0%). m.p.: 284–286 °C. ^1^H-NMR (DMSO-*d*_6_), δ: 12.65 (d, 1H), 8.24 (d, *J* = 6.9 Hz, 2H), 7.81 (d, *J* = 8.3 Hz, 2H), 7.69 (s, 1H), 7.56 (dd, *J* = 16.6, 8.3 Hz, 4H), 7.38 (d, 2H), 3.64 (d, 4H), 3.38 (m, 4H), 3.18 (s, 2H). ESI-HRMS: *m*/*z* [M + H]^+^, calculated for C_25_H_22_N_6_O_2_S: 471.1603; found: 471.1596.

*2-(4-(4-(4-Methylbenzyl)piperazine-1-carbonyl)phenyl)-1H-thieno[3,4-d]imidazole-4-carboxamide* (**13i**). The title compound was obtained similarly to **13f** as a white solid (0.15 g, 58.0%). m.p.: 272–273 °C. ^1^H-NMR (DMSO-*d*_6_), δ: 12.65 (s, 1H), 8.30 (d, *J* = 8.3 Hz, 2H), 7.84 (d, 2H), 7.78 (s, 1H), 7.58 (m, 4H), 7.35 (m, 2H), 3.87 (s, 3H), 3.43–3.58 (m, 8H), 2.45 (s, 3H). ESI-HRMS: *m*/*z* [M + H]^+^, calculated for C_25_H_25_N_5_O_2_S: 460.1807; found: 460.1809.

*2-(4-(4-(Cyclopropanecarbonyl)piperazine-1-carbonyl)phenyl)-1H-thieno[3,4-d]imidazole-4-carboxamide* (**13j**). The title compound was obtained similarly to compound **13f** as a white solid (0.15 g, 58.0%). m.p.: 272–273 °C. ^1^H-NMR (DMSO-*d*_6_), δ: 12.64 (d, 2H), 8.25 (dd, *J* = 16.1, 8.0 Hz, 2H), 7.75–7.25 (m, 5H), 3.65 (d, 8H), 1.99 (s, 1H), 0.75 (m, 4H). ESI-HRMS: *m*/*z* [M + H]^+^, calculated for C_21_H_21_N_5_O_3_S: 424.1443; found: 424.1441.

#### 3.2.2. Synthesis of Compounds **16a**–**n**

*Methyl 2-phenyl-1H-thieno[3,4-d]imidazole-4-carboxylate* (**14a**). To a solution of 5 (1.7 g, 9.9 mmol) in 1,4-dioxane (35 mL) was added benzaldehyde (1.9 g, 11.0 mmol). The mixture was stirred at room temperature for 4 h and then heated up to 55 °C. Iodobenzene diacetate (6.4 g, 19.9 mmol) was added and the reaction mixture was stirred for 5 min. After reaction, saturated Na_2_S_2_O_3_ and NaHCO_3_ solution was added and the mixture was extracted with DCM (3 × 40 mL). The combined organic was washed with water, brine, dried over Na_2_SO_4_ and purified by column chromatography (PE:EA = 5:1) to afford **14a** as a white solid (0.85 g, 32.9%). ESI-MS *m/z*: 259.3 [M + H]^+^.

*2-Phenyl-1H-thieno[3,4-d]imidazole-4-carboxylic acid* (**15a**). Intermediate **14a** (0.85 g, 3.3 mmol) was added into a 50 mL round-bottom flask and dissolved in CH_3_OH (25 mL), then 4N NaOH solution (7 mL) was added and the mixture was stirred at reflux overnight. After reaction, the pH was adjusted to 6–7 with 2N HCl. The resulting white solid was separated by filtration and used for the next step without further purification (0.68 g, 84.6%).

*2-Phenyl-1H-thieno[3,4-d]imidazole-4-carboxamide* (**16a**). Compound **15a** (0.68 g, 2.8 mmol), CDI (0.45 g, 9.2 mmol) was dissolved in DMF (10 mL) and the mixture was stirred at room temperature for 2 h. Then the solution was added dropwise to aqueous ammonia (45 mL) and stirred overnight. The mixture was extracted with 60 × 3 EA and the combined organic layer was washed with brine, dried with anhydrous Na_2_SO_4_ and concentrated *in vacuo*. The crude product was recrystallized with CH_3_OH to give **16a** as a white solid (0.15 g, 22.1%). m.p.: 256–257 °C. ^1^H-NMR (DMSO-*d*_6_), δ: 12.54 (d, 1H), 8.18 (m, 2H), 7.69 (s, 1H), 7.62–7.55 (m, 3H), 7.52 (s, 1H), 7.35 (s, 1H). ^13^C-NMR (DMSO-*d*_6_), δ: 162.66, 150.60, 139.05, 131.22, 129.53, 129.09, 127.20, 113.82, 110.20, 101.06. ESI-HRMS: *m*/*z* [M + H]^+^, calculated for C_12_H_9_N_3_OS: 244.0544, found: 244.0539.

*2-(4-Fluorophenyl)-1H-thieno[3,4-d]imidazole-4-carboxamide* (**16b**). The title compound was obtained similarly to **16a** as a white solid (0.18 g, 20.9%). m.p.: 244–246 °C. ^1^H-NMR (DMSO-*d*_6_), δ: 12.75 (d, 1H), 8.29–8.17 (m, 2H), 7.63 (d, 1H), 7.44 (m, 3H), 7.34 (d, 1H). ESI-HRMS: *m*/*z* [M + H]^+^, calculated for C_12_H_8_FN_3_OS: 262.0450, found: 262.0451.

*2-(4-Chlorophenyl)-1H-thieno[3,4-d]imidazole-4-carboxamide* (**16c**). The title compound was obtained similarly to **16a** as a white solid (0.14 g, 16.5%). m.p.: 245–246 °C. ^1^H-NMR (DMSO-*d*_6_), δ: 12.63 (d, 1H), 8.13–8.27 (m, 2H), 7.59–7.66 (m, 2H), 7.33–7.49 (m, 3H). ESI-HRMS: *m*/*z* [M + H]^+^, calculated for C_12_H_8_ClN_3_OS: 278.0155, found: 278.0152.

*2-(2-Chlorophenyl)-1H-thieno[3,4-d]imidazole-4-carboxamide* (**16d**). The title compound was obtained similarly to **16a** as a white solid (0.10 g, 13.2%). m.p.: 202–204 °C. ^1^H-NMR (DMSO-*d*_6_), δ: 12.50 (d, 1H), 7.85 (d, *J* = 6.7 Hz, 1H), 7.72–7.64 (m, 2H), 7.64–7.50 (m, 2H), 7.49–7.21 (m, 2H). ^13^C-NMR (DMSO-*d*_6_), δ: 162.56, 160.56, 151.70, 149.95, 138.73, 132.10, 130.51, 129.37, 127.47, 114.40, 110.81, 101.67. ESI-HRMS: *m*/*z* [M + H]^+^, calculated for C_12_H_8_ClN_3_OS: 278.0155, found: 278.0148.

*2-(2,6-Dichlorophenyl)-1H-thieno[3,4-d]imidazole-4-carboxamide* (**16e**). The title compound was obtained similarly to compound **16a** as a white solid (0.12 g, 15.4%). Mp: 210–212 °C. ^1^H-NMR (DMSO-*d*_6_), δ: 12.56 (d, 1H), 7.88–7.74 (m, 3H), 7.65 (t, *J* = 7.8 Hz, 1H), 7.47–7.38 (m, 2H). ESI-HRMS: *m*/*z* [M + H]^+^, calculated for C_12_H_7_Cl_2_N_3_OS: 311.9765, found: 311.9767.

*2-(4-(Trifluoromethyl)phenyl)-1H-thieno[3,4-d]imidazole-4-carboxamide* (**16f**). The title compound was obtained similarly to compound **16a** as a white solid (0.15 g, 22.6%). m.p.: 283–285 °C. ^1^H-NMR (DMSO-*d*_6_), δ: 12.78 (d, 1H), 8.40 (d, *J* = 8.3 Hz, 2H), 7.96 (d, *J* = 8.3 Hz, 2H), 7.79–7.27 (m, 3H). ^13^C-NMR (DMSO-*d*_6_), δ: 162.79, 161.13, 139.35, 133.87, 131.03, 128.30, 126.47, 125.86, 123.14, 102.05. ESI-HRMS: *m*/*z* [M + H]^+^, calculated for C_13_H_8_F_3_N_3_OS: 312.0418, found: 312.0414.

*2-(3-(Trifluoromethyl)phenyl)-1H-thieno[3,4-d]imidazole-4-carboxamide* (**16g**). The title compound was obtained similarly to compound **16a** as a white solid (0.14 g, 21.8%). m.p.: 240–241 °C. ^1^H-NMR (DMSO-d_6_), δ: 12.79 (d, 1H), 8.56 (s, 1H), 8.50 (t, *J* = 10.9 Hz, 1H), 7.94 (t, *J* = 7.4 Hz, 1H), 7.83 (t, *J* = 7.8 Hz, 1H), 7.74–7.33 (m, 3H). ^1^H-NMR (DMSO-*d*_6_, D_2_O), δ: 8.45–8.54 (m, 2H), 7.96 (d, *J* = 7.9 Hz, 1H), 7.85 (t, *J* = 7.9 Hz, 1H), 7.49 (s, 1H). ^13^C-NMR (DMSO-*d*_6_), δ: 162.93, 161.39, 152.06, 150.58, 139.41, 131.56, 130.73, 128.05, 124.07, 114.94, 110.86, 101.91. ESI-HRMS: *m*/*z* [M + H]^+^, calculated for C_13_H_8_F_3_N_3_OS: 312.0418, found: 312.0413.

*2-(2-(Trifluoromethyl)phenyl)-1H-thieno[3,4-d]imidazole-4-carboxamide* (**16h**). The title compound was obtained similarly to compound **16a** as a yellow solid (0.14 g, 32.5%). m.p.: 241–243 °C. ^1^H-NMR (DMSO-d_6_), δ: 12.57 (d, 1H), 7.97 (t, *J* = 9.4 Hz, 1H), 7.91–7.75 (m, 3H), 7.65 (s, 1H), 7.40 (s, 1H), 7.20 (s, 1H). ^13^C-NMR (DMSO-*d*_6_), δ: 162.95, 161.28, 150.69, 138.99, 133.08, 132.27, 131.54, 129.62, 128.35, 127.26, 125.52, 122.80, 114.84, 111.14, 102.11. ESI-HRMS: *m*/*z* [M + H]^+^, calculated for C_13_H_8_F_3_N_3_OS: 312.0418, found: 312.0413.

*2-(4-Nitrophenyl)-1H-thieno[3,4-d]imidazole-4-carboxamide* (**16i**). The title compound was obtained similarly to compound **16a** as a yellow solid (0.12 g, 24.7%). m.p.: 278–279 °C. ^1^H-NMR (DMSO-*d*_6_), δ: 12.89 (d, 1H), 8.52–8.34 (m, 4H), 7.73–7.44 (m, 3H). ^13^C-NMR (DMSO-*d*_6_), δ: 162.85, 160.65, 150.62, 149.14, 139.36, 135.78, 128.80, 124.71, 115.40, 111.84, 102.24. ESI-HRMS: *m*/*z* [M + H]^+^, calculated for C_12_H_8_N_4_O_3_S: 289.0395, found: 289.0392.

*2-(4-Methoxyphenyl)-1H-thieno[3,4-d]imidazole-4-carboxamide* (**16j**). The title compound was obtained similarly to compound **16a** as a white solid (0.16 g, 45.2%). m.p.: 240 °C–241 °C. ^1^H-NMR (DMSO-*d*_6_), δ: 12.53 (d, 1H), 8.14 (d, *J* = 8.9 Hz, 2H), 7.47 (m, 2H), 7.27 (s, 1H), 7.13 (d, *J* = 8.9 Hz, 2H), 3.86 (s, 3H). ^13^C-NMR (DMSO-*d*_6_), δ: 163.19, 160.12, 151.12, 139.51, 131.17, 130.67, 120.04, 117.59, 112.68, 101.45, 55.17. ESI-HRMS: *m*/*z* [M + H]^+^, calculated for C_13_H_11_N_3_O_2_S: 274.0650, found: 274.0651.

*2-(3-Methoxyphenyl)-1H-thieno[3,4-d]imidazole-4-carboxamide* (**16k**). The title compound was obtained similarly to compound **16a** as a white solid (0.15 g, 38.2%). m.p.: 230–242 °C. ^1^H-NMR (DMSO-*d*_6_), δ: 12.52 (d, 1H), 7.76 (d, *J* = 7.4 Hz, 2H), 7.46 (m, 4H), 7.14 (d, *J* = 8.0 Hz, 1H), 3.87 (s, 3H). ^13^C-NMR (DMSO-*d*_6_), δ: 163.19, 159.98, 150.97, 139.42, 131.17, 130.67, 120.04, 117.59, 112.68, 101.24, 55.88. ESI-HRMS: *m*/*z* [M + H]^+^, calculated for C_13_H_11_N_3_O_2_S: 274.0650, found: 274.0648.

*2-(p-Tolyl)-1H-thieno[3,4-d]imidazole-4-carboxamide* (**16l**). The title compound was obtained similarly to compound **16a** as a white solid (0.10 g, 19.8%). m.p.: 257–258 °C. ^1^H-NMR (DMSO-*d*_6_), δ: 12.65 (s, 1H), 8.08 (m, 2H), 7.66 (s, 1H), 7.53 (s, 1H), 7.39 (d, *J* = 8.1 Hz, 2H), 7.31 (s, 1H), 2.40 (s, 3H). ^13^C-NMR (DMSO-*d*_6_), δ: 163.24, 151.18, 141.48, 139.48, 137.64, 130.04, 127.64, 113.79, 110.38, 101.27, 21.58. ESI-HRMS: *m*/*z* [M + H]^+^, calculated for C_13_H_11_N_3_OS: 258.0701, found: 258.0698.

*2-(m-Tolyl)-1H-thieno[3,4-d]imidazole-4-carboxamide* (**16m**). The title compound was obtained similarly to compound **16a** as a white solid (0.12 g, 26.2%). m.p.: 254–255 °C. ^1^H-NMR (DMSO-*d*_6_), δ: 12.65 (s, 1H), 8.03 (s, 1H), 7.97 (d, *J* = 7.7 Hz, 1H), 7.63 (s, 1H), 7.46 (t, *J* = 7.6 Hz, 2H), 7.39 (d, *J* = 7.5 Hz, 2H), 2.42 (s, 3H). ^13^C-NMR (DMSO-*d*_6_), δ: 163.03, 151.00, 139.41, 138.83, 132.30, 129.90, 129.40, 128.07, 124.78, 113.98, 110.50, 101.35, 21.51. ESI-HRMS: *m*/*z* [M + H]^+^, calculated for C_13_H_11_N_3_OS: 258.0701, found: 258.0696.

*2-(o-Tolyl)-1H-thieno[3,4-d]imidazole-4-carboxamide* (**16n**). The title compound was obtained similarly to compound **16a** as a white solid (0.14 g, 31.5%). m.p.: 222–223 °C. ^1^H-NMR (DMSO-*d*_6_), δ: 12.43 (s, 1H), 7.75 (d, *J* = 7.4 Hz, 1H), 7.63 (d, *J* = 12.3 Hz, 1H), 7.28–7.46 (m, 5H), 2.61 (s, 3H). ^13^C-NMR (DMSO-*d*_6_), δ: 163.98, 163.14, 152.79, 151.03, 138.94, 138.08, 131.93, 130.86, 130.00, 126.58, 114.24, 110.84, 101.45, 21.54. ESI-HRMS: *m*/*z* [M + H]^+^, calculated for C_13_H_11_N_3_OS: 258.0701, found: 258.0700.

### 3.3. PARP-1 Enzymatic Inhibition Assay

We used a commercially available 96-well assay kit (Cat# 4690-096-K, Trevigen, Gaithersburg, MD, USA) to evaluate the inhibitory activity. The procedure was according to the instructions provided by the manufacturer. The stock solutions of various test compounds were dissolved in DMSO and serially diluted to the required concentrations. 25 μL of 1 × Buffer was added to each well and 25 μL of appropriate 2 × NAD^+^ standards were added to 1B to 1G and 2B to 2G well of 96-well plate for standard curve. 25 μL of 2 μM NAD was added to 1H and 2H for PARP control. 25 μL of 2 μM NAD was added to wells for test compounds, then serially diluted compounds were added, and 1 μL of DMSO was added to the standard well. 25 μL of PARP Mix minus enzyme was added to 1A to 1G and 25 μL of PARP Mix plus enzyme was added to PARP control and compounds well. Veliparib and olaparib were used as control compounds. The 96-well plate was incubated at 25 °C for 30 min, then 50 μL Cycling Mix was added to all wells and mixed with pipette. Incubate the 96-well plate in the dark for 40 min at 25 °C. Then 50 μL stop solution was added to each well, and mixed with pipette. Fluorescence values were measured under the conditiond of 544 nm excitation wavelength and 590 nm emission wavelength. Then draw the standard curve and calculate the inhibition rate of each test compound. IC_50_ value of each compound was calculated according to the above results.

### 3.4. CellTiter Glo Assay for Cell Growth Inhibition

The growth inhibition potential of test compounds was determined on BRCA1-deficient cell lines HCC1937 and BRCA2-deficient cell lines CAPAN-1. The stock solutions of test compounds were dissolved in DMSO and serially diluted to the required concentrations for 9 doses. Cells were treated with compounds with duplications and incubated for 96 h at 37 °C under 5% CO_2_ atmosphere. 60 μL medium was removed from each well and plates were kept at room temperature for 30 min. 60 μL reagent (Celltiter Glo assay kit, Promega, Sunnyvale, CA, USA) per well was added and plates were shaked for 2 min. Cells were incubated avoiding light at room temperature for 30 min and the luminescence value was recorded by multiplate reader. GraphPad Prism 5 was used to plot effect-dose curves and calculate the IC_50_ values of compounds.

### 3.5. Cytotoxicity Assay on Normal Human Lung Fibroblast (HLF) Cells

The cytotoxic potencies of test compounds were determined on normal human lung fibroblast (HLF) cells. Cells were seeded into 96-well plate at 3000 cells/well. The stock solutions of test compounds were dissolved in DMSO and serially diluted to the required concentrations for 7 doses, and the final testing concentrations for these compounds were 0.3, 1, 3, 10, 30, 100, 300 µM. Cells were treated with compounds with triplications and incubated for 72 h at 37 °C under 5% CO2 atmosphere. The fraction of surviving cells after compound treatment was determined using the MTT assay. IC_50_ is defined as the drug concentration producing 50% decrease of cell growth.

### 3.6. ADMET Prediction

ADMET studies were performed *in silico* by using ADMET predictor 7.0 (Simulations Plus). The input files containing structures of testing compounds were prepared by Chemdraw 14.0 and saved in SDF format. These files were uploaded into the ADMET predictor software for further evaluations. Various pharmacokinetic parameters like logP and logD, human intestinal absorption, plasma protein binding (PPB), *etc.* were estimated for all compounds at pH 7.4. The output files display lists of compounds, ADMET properties and molecular discriptors in tab- delimited tabular format.

### 3.7. Docking

To prepare the receptor, the PDB file (PDB code: 2RD6) was downloaded and dealt with using Discovery Studio 3.0. Hydrogen atoms and electric charges were added after deleting the protein water molecules. Then residues within 5 Å of A861695 were selected and the sphere from selection prepared. To prepare the ligand the structure (**16l**) was drawn with Chemdraw 12.0 and the molecular energy minimized using the function (generate conformations). For molecular docking the CDocker protocols are run and the best confirmation selected and hydrogen bond interactions and π-π interactions display in the 2D diagram. The 3D image was presented with PyMOL.

## 4. Conclusions

In summary, this study reports the design, synthesis, and biological activity of novel 1*H*- thieno[3,4-*d*]imidazole-4-carboxamide derivatives as PARP-1 inhibitors. The preliminary enzymatic inhibitory activity of compounds **13a**–**j** indicated that the large piperazine-containing side chains could not be tolerated in the thienoimidazole scaffold, but this also provided a basis for further designing PARP-1 inhibitors. The newly synthesized compounds **16a**–**n** with short side chains displayed good enzymatic activities, and among them, **16l** was the most potent one with activity comparable to olaparib. Cellular evaluations indicated that the anti-proliferative activity of **16g**, **16i**, **16j** and **16l** against BRCA-deficient cell lines was similar to that of olaparib, while the cytotoxicity of **16j** and **16l** toward human normal cells was lower. Furthermore, the ADMET prediction results indicated that these compounds might possess better toxic and pharmacokinetic properties. Further evaluation of the pharmacokinetic studies and anti-proliferative activities is on-going and will be reported in the near future.

## Figures and Tables

**Table 1:**
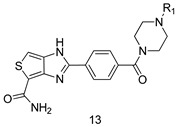
Structure and PARP-1 enzyme inhibitory activity of compounds **12** and **13a**–**j**. ^a^

Compd.	R_1_	IC_50_ (μM)	Compd.	R_1_	IC_50_ (μM)
Olaparib		0.018	**13f**	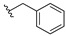	3.864
Veliparib		0.005	**13g**	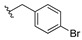	ND
**13a**	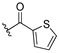	2.569	**13h**	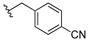	3.724
**13b**	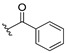	3.763	**13i**	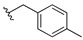	ND
**13c**	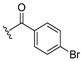	1.874	**13j**	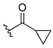	1.525
**13d**	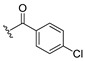	ND ^b^	**12**	H	0.723
**13e**	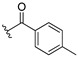	ND			

^a^: IC_50_ values were calculated by logit method from the results using six concentrations for each compound. ^b^: ND: not determined. IC_50_ of the compounds with <50% PARP-1 inhibitory ratios at 10 μM was not determined.

**Table 2:**
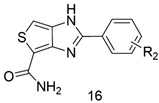
The structures and PARP-1 enzyme inhibitory activity of compounds **16a**–**n**. ^a^

Compd.	R_2_	IC_50_ (μM)	Compd.	R_2_	IC_50_ (μM)
Olaparib		0.013	**16g**	3-CF_3_	0.151
Veliparib		0.014	**16h**	2-CF_3_	ND
**16a**	H	0.827	**16i**	4-NO_2_	0.136
**16b**	4-F	0.738	**16j**	4-OCH_3_	0.102
**16c**	4-Cl	0.354	**16k**	3-OCH_3_	1.491
**16d**	2-Cl	1.488	**16l**	4-CH_3_	0.043
**16e**	2-Cl, 5-Cl	ND ^b^	**16m**	3-CH_3_	5.787
**16f**	4-CF_3_	0.548	**16n**	2-CH_3_	0.328

^a^: IC_50_ values were calculated by a logit method from the results using six concentrations for each compound. ^b^: ND: not determined. IC_50_ of the compounds with <50% PARP-1 inhibitory activity at 10 μM was not determined.

**Table 3:** Anti-proliferative activity of compounds against BRCA-1 and BRCA-2 deficient cell lines. ^a,b^

Compd.	IC_50_ (μM) (BRCA-1, HCC1937)	IC_50_ (μM) (BRCA-2, CAPAN-1)
Olaparib	132.92 ± 14.39	13.36 ± 1.16
Veliparib	127.04 ± 18.10	57.67 ± 12.39
**16g**	31.34 ± 0.35	38.63 ± 6.75
**16i**	32.87 ± 4.21	26.79 ± 1.55
**16j**	55.14 ± 0.24	46.60 ± 0.62
**16l**	49.25 ± 5.43	38.77 ± 1.60

^a^: Anti-proliferative activity of compounds was evaluated in HCC1937 and CAPAN-1 cell lines using CellTiter Glo Assay. ^b^: IC_50_ values were calculated by a logit method from the results using nine concentrations for each compound.

**Table 4:** Selected ADMET properties of compounds.

ADMET Properties	AZD-2281	16g	16i	16j	16l
S + logP	2.26	2.6	1.7	1.98	1.3
S + Sw (mg/mL)	2.0 × 10^−3^	7.86 × 10^−3^	4.69 × 10^−3^	2.16 × 10^−2^	1.03 × 10^−2^
S + FaSSGF (mg/mL)	1.40 × 10^−2^	3.50 × 10^−1^	8.85 × 10^−2^	3.68 × 10^−1^	4.19 × 10^−1^
S + Peff (×10^4^ cm/s)	3.26	3.18	1.59	1.86	2.32
S + BBB_Filter	Low	High	High	High	High
Absn_risk	0.17	0.31	0.76	0.0	0.0
CYP3A4_substr	Yes	NO	NO	NO	NO
CYP_risk	1.95	0	0	0	0
Rat acute toxicity (LD_50_; mg/kg)	380.03	240.10	1550.48	1741.25	1520.52
Tox_risk	2.73	2.67	3	1	1
ADMET_risk	4.85	3.02	3.76	1	1

S + logP: octanol-water partition coefficient, the logP values of drugs mostly range from −2.5 to 5.5. S + Sw: native water solubility. S + FaSSGF: solubility in simulated gastrointestinal fluids. S + Peff: human jejunal effective permeability. S+BBB_Filter: qualitative likelihood (High/Low) of crossing the blood-brain barrier. Absn_risk: ADMET risk for oral absorption in human. CYP3A4_ substr: qualitative assessment of a molecule being the substrate of CYP 1A2 in human. CYP_risk: ADMET risk for metabolic liability. Tox_risk: ADMET risk for toxic liability. ADMET_risk: the overall evaluation of ADMET predictor 7.0.

**Table 5:** The results of cytotoxicity assay on normal human lung fibroblast cells. ^a,b^

Compd.	Inhibition Rate (%)							IC_50_ (µM)
	0.3 µM	1 µM	3 µM	10 µM	30 µM	100 µM	300 µM	
Olaparib	1.88	1.83	1.10	1.37	1.04	29.60	91.45	134.30 ± 3.14
Veliparib	1.00	−0.28	−1.20	−1.35	−0.06	−0.45	93.64	~200
**16g**	−1.65	−1.02	−0.63	0.76	4.13	69.88	99.40	77.63 ± 1.50
**16i**	−0.05	1.33	0.99	3.37	2.56	55.26	99.63	94.11 ± 2.20
**16j**	0.68	2.85	2.68	6.95	9.43	12.63	32.37	>300
**16l**	0.42	3.80	7.12	10.16	13.65	23.20	27.94	>300

^a^: Cytotoxicity of compounds was evaluated in HLF cell line using MTT assay. ^b^: Cells were treated with compounds in triplicate for 72 h.
